# Multiscale Mechanisms of Exercise-Induced Neuroplasticity: From Molecular Pathways to Network Dynamics and Behavioral Adaptation

**DOI:** 10.3390/brainsci16030294

**Published:** 2026-03-06

**Authors:** Xue Wang, Jun Zhang, Xiaoyu Wang, Shuren Wang, Yidan Zhang, Yupeng Yang, Xuchang Zhou, Chang Liu, Junjie Liu, Mi Zheng

**Affiliations:** 1 Graduate School, Harbin Sport University, Harbin 150008, China; 15515077401@163.com (X.W.);; 2 Department of Rehabilitation Medicine, The First Affiliated Hospital of Xiamen University, School of Medicine, Xiamen University, Xiamen 361003, China; 3 School of Sport Medicine and Rehabilitation, Beijing Sport University, Beijing 100084, China; 4 School of Basic Medical Sciences, China Three Gorges University, Yichang 443002, China; 5 College of Science and Technology, China Three Gorges University, Yichang 443002, China

**Keywords:** exercise, neuroplasticity, BDNF, brain networks, cognitive function, behavioral adaptation, dose–response, individual differences, translational neuroscience

## Abstract

**Highlights:**

**What are the main findings?**

- Exercise promotes neuroplasticity through a multiscale mechanism, integrating molecular pathways like BDNF signaling and mitochondrial adaptation with large-scale functional brain network reorganization.
- Distinct neural mechanisms underlie acute versus chronic exercise, where acute bouts trigger immediate neurochemical modulation while chronic training induces long-term structural remodeling and network homeostasis.

**What are the implications of the main findings?**

- Physical activity acts as a “multi-target” behavioral intervention capable of enhancing executive function, regulating emotions, and aiding rehabilitation in addiction and neurodegenerative disorders.
- To maximize therapeutic efficacy, clinical applications should adopt “precision exercise prescriptions” that account for individual variability in genetics, environment, and dose–response relationships.

**Abstract:**

Exercise as a non-pharmacological measure is important to increase the brain plasticity hence improving cognitive performance as well as mental health. This narrative review describes in depth the hierarchical multiscale processes of neuroplasticity to exercise, including the presence of neurotrophic factor regulation, cellular metabolic adaptations and neurotransmitter remodeling, up to the structure and functional reorganization of brain networks as seen through neuroimaging, and concluding with adaptive cognitive and behavioral outcomes. We further investigate the role of personal variations in genetic time and social environments in moderating the neuroplasticity of exercise. Furthermore, the review identifies the importance of combining multimodal visualization methods with computational models in generating accurate workout prescriptions and their potential of translation into clinical and educational practice. Lastly, the research problems and “grand challenges” are addressed, with a focus on the importance of exercise as a pleiotropic behavior-intervention and its general implications to the area of promoting brain health.

## 1. Introduction

It has been acknowledged for a long time that physical activity is advantageous to the functioning of the brain, cognitive performance, and mental health at all life stages [1]. However, a critical distinction must be made between physical activity (PA), defined as any energy-expending bodily movement, and physical exercise, which represents a planned, structured subcategory of PA aimed at improving physical fitness. A significant amount of epidemiological and experimental studies have established that physical exercise offers cognitive strength and mental health between childhood and old age [2,3,4]. To illustrate, physical exercise activities have been demonstrated to have a decisive role in the alleviation of depression and anxiety symptoms, especially in the quality of sleep and the reduction in sedentary habits during the COVID-19 lockdowns, which subsequently positively affected mental health outcomes [5]. Equally, studies of an international nature such as the Collaborative outcomes study on Health and Functions during Infection Times (COHFIT) where lifestyle determinants which could be modified, such as physical activity, were the center of focus when it comes to the enhancement of mental health in crisis situations [6]. These findings underscore the extensive implications of exercise on the wellbeing of the brain and psychological adaptation, and it can be among the most significant adjustable variables in maintaining the psychological and emotional functions of the body in a multitude of situations.

Physical activity produces its neuroprotective and neuroplastic effects in complex mechanisms that have biological effects at a variety of biological scales. Exercise can affect the neurotrophic factors, neurotransmitter systems and metabolic pathways at both molecular and cellular levels to enhance neuroplasticity and neurogeneration at the synapses [7]. While rodent models provide high-resolution mechanistic insights into these molecular pathways, the translational verification in humans remains a significant challenge, often relying on peripheral biomarkers that may not fully reflect central neural adaptations. As an illustration, a study on blood-circulating biomarkers of brain health in overweight or obese children indicated that despite the lack of significant changes in the classic candidate biomarkers of brain-derived neurotrophic factor or 2-hydroxybutyrate, exercise decreased the levels of macrophage scavenger receptor type-I, implying new directions through which exercise can be used to regulate brain health [6]. In addition to molecular alterations, exercise also changes brain network dynamics and connectivity, which is demonstrated by neuroimaging experiments of the brain activity of functional brain networks. Similar to exercise-induced neuroplasticity, brief mindfulness and focused attention practices have been found to alter large-scale brain networks that are devoted to cognitive control as well as salience processing, but more behaviorally relevant aspects of them still need to be clarified [8]. Furthermore, studies of stress and isolation conditions, including Antarctic programs, indicate that exercise and sleep over also are capable of averting temporary gray matter atrophy, highlighting the connection between lifestyle aspects and brain standards [9].

Furthermore, the impact on mental health and brain function is inherently positive, and it is manifested in the ability to modulate behavior and network-level changes that facilitate adaptations in cognitive and emotional resilience [10]. Exercise induces adaptive adjustments that help in effective processing of information, regulation of emotions and behavioral flexibility, which is essential in the management of environmental issues and stressors [11]. Notably, these effects are not general but individual, and variability depends on genetic, developmental and environmental factors. For example, depending on sex hormones, interactions between hosts and microbiomes of the organism are relevant to cardiovascular and, possibly, neurocognitive health, which means that endocrine and microbial factors can be used to mediate personal responses to physical activity and other lifestyle changes [12]. Moreover, sociodemographic (e.g., level of technological skills, age, and distance from the clinic) and lifestyle factors (e.g., physical activity, diet) influence preferences toward in- and remote-based cognitive rehabilitation, which implies that individual-based interventions should maximize the effectiveness of the interventions [13].

Considering the multiscale nature and complexity of exercise-induced neuroplasticity, it is justified to synthesize the underlying mechanisms, from the molecular signaling pathways scale to high-scale brain network dynamics and behavior change responses [14]. It is the purpose of this review to synthesize the existing evidence on the multiscale processes through which exercise produces neural plasticity including acute and chronic exercise effects, molecular and neuroimaging biomarkers, the dose–response relation, and the translational application of this topic [15,16,17]. In explaining the particularities of the exercise responses in subjects and the subject-specific variability, we would like to offer a theoretical framework to contribute to future studies and clinical application towards the possibility of using physical activity to promote mental health and stimulate it otherwise during the lifespan [18]. Based on the existing literature, we propose an integrative conceptual model hypothesizing that exercise-induced neuroplasticity is a hierarchical regulatory process. This process initiates with molecular triggers (e.g., BDNF, mitochondrial signaling), translates into structural remodeling (e.g., white matter integrity), and culminates in functional brain network reorganization that drives adaptive behavioral outcomes. This multiscale framework serves as the core hypothesis guiding this review. Moving beyond a descriptive overview, this synthesis identifies the ‘grand challenges’ of the field, specifically focusing on the translation of molecular findings to clinical application, the research gaps in personalized exercise prescriptions, and the scalability of these interventions.

## 2. Search Methodology

### 2.1. Literature Search Strategy and Selection Criteria

This study was designed as a narrative review aimed at synthesizing the multiscale mechanisms of exercise-induced neuroplasticity. We conducted a comprehensive search of the literature across major electronic databases, including PubMed, Web of Science, Scopus, and Google Scholar, covering the period from inception up to December 2025. The search strategy employed combinations of the following medical subject headings (MeSH) and free-text terms: “physical exercise,” “neuroplasticity,” “BDNF,” “mitochondrial adaptation,” “brain networks,” “cognitive function,” and “synaptic plasticity.” Inclusion criteria prioritized peer-reviewed original research and meta-analyses that provided mechanistic insights into molecular, structural, or functional neural adaptations in both rodent models and human clinical trials. While this work does not follow the PRISMA guidelines for systematic reviews, studies were critically selected based on their methodological rigor, novelty, and relevance to the proposed multiscale theoretical framework. We acknowledge the inherent limitations of this narrative approach, including the potential for selection bias and the absence of quantitative meta-analytic synthesis. To mitigate these biases, we prioritized high-quality evidence from randomized controlled trials and longitudinal studies where available.

### 2.2. Methodological Limitations of Reviewed Studies

It is crucial to critically assess the quality of the evidence synthesized in this review. Preclinical studies, while providing mechanistic depth, often rely on small sample sizes (frequently *n* < 10 per group), which may limit statistical power and reproducibility. Conversely, human clinical trials face inherent challenges regarding confounding factors (e.g., diet, sleep, baseline fitness) and the difficulty of blinding participants to exercise interventions. Furthermore, “ceiling effects” in cognitive tasks are often observed in healthy young adults, potentially masking the benefits of exercise that are more apparent in aging or clinical populations. These methodological constraints necessitate a cautious interpretation of the translational potential of current findings.

## 3. Molecular and Cellular Processes of Exercise-Induced Neural Plasticity

### 3.1. Neurotrophic Factors and Synaptic Plasticity

Exercise manifests itself with a robust increase in essential neurotrophic factors, including brain-derived neurotrophic factor (BDNF), insulin-like growth factor-1 (IGF-1), and vascular endothelial growth factor (VEGF) [19]. These factors support neuronal survival, synaptic formation, and neurogenesis, and thus form the basis of exercise-receptor neural plasticity [20]. Experiments on animal models show that with the combination of exercise and lutein/zeaxanthin intervention, the levels of BDNF and synaptic proteins such as synapsin I, synaptophysin (SYP) and growth-associated protein 43 (GAP-43) increase significantly in the cerebral cortex and provide indications of greater synaptic remodeling and neuroprotection [21]. The BDNF–TrkB receptor mechanism is at the core of this mechanism; this activation of TrkB by BDNF activates long-term potentiation (LTP) and synaptic strength that depends on downstream signaling cascade activation of MAPK and PI3K/Akt pathways [22]. BDNF expression in models of ischemic stroke rapidly increases in response to stroke, and has been shown to modulate markers of synaptic plasticity including SYP, PSD-95 and MAP-2, and inhibition of BDNF–TrkB signaling inhibits recovery at the synapse where this system is expressed [23]. IGF-1 and VEGF are complementary in terms of their angiogenesis and metabolic support, and responses to the brain to enhance its adaptive remodeling; exercise raises their serum and brain concentrations, which are associated with better age-related and disease-related cognitive functions [24,25]. The clinical research of mild cognitive impairment (MCI) patients has shown that interval training (specifically high-intensity interval training, HIIT) elevates serum BDNF, IGF-1 and VEGF, and synaptic plasticity, which is measured by means of transcranial magnetic stimulation, also improves [26]. Furthermore, up-regulation of neurotrophic factors has been found to be exercise-induced in models of asthma, depression, Alzheimer’s disease (AD), and traumatic brain injury (TBI), and has an overall neuroprotective effect. Functional and structural synaptic plasticity is also mediated by modulation of synaptic proteins and neurotransmitter receptors by BDNF and other neurotrophic factors, as demonstrated by up-regulation of synaptophysin and postsynaptic membrane protein density 95 (PSD-95) following exercise or neurotrophic factor treatment [27,28]. Although clinical trials conducted on Parkinson disease (PD) show no significant alterations in serum neurotrophic factors following combinations of aerobic and task-centered experiences, preclinical results substantiate the possibilities of exercise in its capacity to increase the BDNF and IGF-1 signaling and sustain synapses [29,30]. However, it is important to acknowledge that some clinical studies have reported null findings or conflicting results regarding serum neurotrophin levels, suggesting that individual baseline fitness and genetic polymorphisms may significantly modulate the neuroplastic response [31]. While BDNF is the most extensively studied neurotrophin, glial cell line-derived neurotrophic factor (GDNF) also plays a critical role, particularly in the survival and maintenance of dopaminergic neurons (Table 1) [32,33]. Although less abundant in exercise literature compared to BDNF, emerging evidence suggests that physical exercise can upregulate GDNF expression in the striatum and hippocampus, thereby supporting motor control and cognitive plasticity [34]. However, it is important to note that the majority of these findings are derived from rodent models, and translational evidence in humans remains limited. Future research is needed to clarify the dose–response relationship between exercise intensity and GDNF secretion in human populations [35,36].

The combination of these results explains a multi-factorial molecular mechanism underlying exercise-induced synaptic plasticity and neurotrophic factors play a central role in the relationship between physical activity and cognitive and neural resilience (Table 1).

### 3.2. Metabolic and Mitochondrial Adaptations

The intense metabolic remodeling in the brain while exercising results in an increase in the efficiency of energy metabolism to sustain the increase in neuronal demand. It entails an elevation of the glycolysis, oxidative phosphorylation, and mitochondrial biogenesis, and increased capacity to counteract oxidative damages, which facilitate neuronal functioning and survival. Metabolomic studies have demonstrated that acute and chronic exercise enhance the intermediate of the tricarboxylic acid (TCA) cycle of succinate and fumarate as well as amino acids and fatty acid metabolites, which reveal that the mitochondrial substrate is utilized more actively [38,39]. Exercise activates other signaling pathways—such as AMPK and PGC-1α—the latter of which controls mitochondrial biogenesis and metabolic plasticity [40,41]. Exercise helps to preserve mitochondrial membrane potential, mitochondrial swelling, and apoptosis in animal models and thus maintains mitochondrial integrity during conditions of stress, e.g., chemotherapy or ischemia [30,37]. The connexin 43 of the mitochondrial membrane has also been reported to be a mediator of mitochondrial metabolism and mitochondrial adaptation to stresses and introduces new molecular players in exercise-induced mitochondrial plasticity [42]. In parallel, exercise controls the neurotransmitter metabolism related to mitochondrial functioning, such as kynurenine catabolism, that helps with energy efficiency and fatigue resistance [43]. The metabolic changes that accompany exercise move to the peripheral tissues and circulatory systems, and thus impact on the neurotrophic factors and dysfunctional targets, which indirectly influence the brain energy metabolism and plasticity [25,44]. Notably, metabolic plasticity and mitochondrial adaptation play a pivotal role in conditions including diabetes, mood disorders, and neurodegeneration, where exercise counterbalances mitochondrial dysfunction and oxidative stress [45]. Altogether, physical activity can improve metabolic homeostasis of the brain by means of coordinated mitochondrial biogenesis and enhanced use of substrates, as well as the activation of neuro-protective signaling pathways, and support neural plasticity and cognition (Table 2).

### 3.3. Neurotransmitter System Regulation

#### 3.3.1. Modulation of Major Neurotransmitter Systems by Exercise

**Figure 1:**
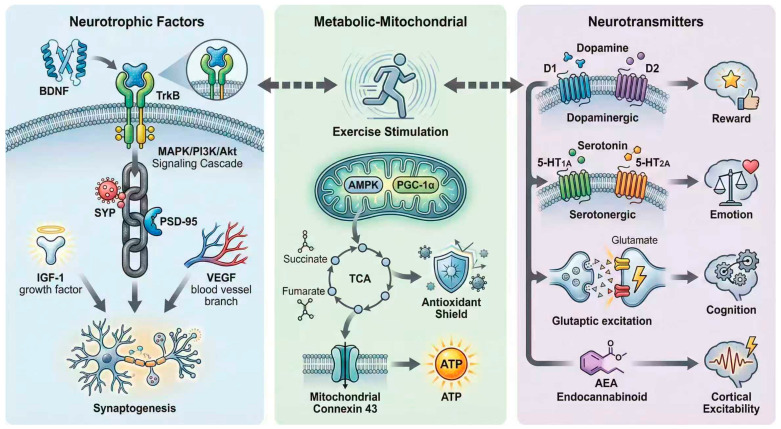
Core mechanisms of exercise-induced neuroplasticity at the molecular-cellular level. Figure 1 centered on “exercise stimulation”, radiating three parallel submodules (neurotrophic factors, metabolic-mitochondrial, neurotransmitters) with dashed arrows indicating cross-regulation.

Exercise is able to regulate various neurotransmitter systems, in particular dopaminergic, glutamatergic, and serotonergic systems, which play a role in the processing of rewards, mood, cognition and motor control. Exercise improves dopamine signaling, which is indicated by the elevation of dopamine levels in the nucleus accumbens and hippocampus, which leads to higher motivation, executive function, and memory [47,48,49]. The locomotor performance and exercise capacity are regulated by exercise-induced stimulation of the dopamine D1 and D2 receptors in the striatum and extra-striatum [25]. The serotonergic system plays a pivotal role as well; exercise not only increases the process of serotonin synthesis and receptor expression, 5-HT2A and 5-HT1A in particular, in the hippocampus and dorsal raphe, but also promotes neurogenesis, synaptic plasticity, and anxiogenic action [50]. Workouts normalize expression of serotonin transporter (SERT), and alter serotonin in peripheral blood mononuclear cells indicating regulation of neurotransmitters throughout the body [30]. Additionally, exercise also affects the glutamate neurotransmission which balances the excitatory–inhibitory signaling and helps to enhance the effectiveness of the synaptic and cognitive capacity [41]. Neurotransmitter systems’ modulation by exercise also overlaps with neurotrophic signaling, with release of BDNF dependent on downstream activities of serotonergic and dopaminergic systems enhancing adaptations by neuroplastic modulations [22,28]. Moreover, exercise affects neuroendocrine pathways, such as the hypothalamic–pituitary–adrenal axis and adenosine signaling, which in turn will vary the neurotransmitter system governing responses to stress and fatigue [51,52]. Drug research demonstrates that exercise may benefit mood and cognition, although there is a tendency to suppress, rather than support, exercise effects by blocking dopamine/serotonin/receptors, which highlights the importance of intact neurotransmitter signaling in exercise effects [53,54]. These data, together with others, indicate that exercise is a powerful stimulator of neurotransmitter systems, which coordinates neurochemical and behavioral changes in the key to mental wellbeing and cognitive processes (Figure 1, Table 3).

#### 3.3.2. Complex Interactions and Conditions of System Prevalence

The regulation of neurotransmitter systems is not an isolated phenomenon but rather a complex interplay where certain systems prevail depending on specific exercise parameters. During acute, high-intensity exercise, the dopaminergic and catecholaminergic systems typically dominate to maintain heightened arousal and manage the physiological stress response [56,57,58]. In contrast, chronic moderate-intensity training tends to shift the balance toward the serotonergic system and neurotrophic pathways, which are essential for promoting emotional homeostasis and long-term neuroplastic resilience [59]. Furthermore, under conditions of extreme physical exertion, the inhibitory signaling of the serotonergic system may override dopaminergic drive, a process central to the mechanisms of “central fatigue”. Recognizing these shifting conditions of prevalence is vital for transitioning from a simple list of effects to a robust analysis of exercise as a multi-target intervention [60,61].

## 4. Exercise-Induced Structural and Functional Brain Plasticity

### 4.1. Structural Brain Changes

Exercise has always been found to cause dramatic structural alteration of the brain, and, more specifically, those areas that have been identified to deal with memory and cognition, including the hippocampus. Neuroimaging evidence which used voxel-based morphometry (VBM) has shown that physical activity induces hippocampal volumes and increases the gray matter density in memory related regions which confirm its neuroprotective effect. As an example, cortical thickening of temporal and occipitotemporal areas, such as the fusiform and lateral occipital cortices, involved in visuomotor integration and action observation, declared in the learning of motor acts, has been linked to exercise interventions involving aerobic exercises in older adults [62,63]. Moreover, according to diffusion tensor imaging (DTI) literature, exercise increases the white matter integrity, particularly in prefrontal cortex and cerebellar activities, by increasing the structural connectivity of neural fiber track. These microstructure improvements help neural network stabilization and performance, which is the basis of thought processes, such as executive control and memory consolidation [64]. It is important to note that these structural changes are not unique to healthy brains but also can be extended to clinical populations; to cite an example, peripheral nerve injury and subsequent treadmill running modify volumetric and microstructural changes in supraspinal areas of the brain including the hippocampus and motor cortex, and recover functional activities [65]. Moreover, neuroplasticity mechanisms’ faculty in exercise is the incorporation of brain-derived neurotrophic factor (BDNF)-signaling, facilitating the synaptic development and neurogenesis also involved in structural remodeling [66]. The overall picture suggests that physical activity plays a vital part in preserving brain performance and resistance to neurodegeneration by providing a biological basis of brain structural changes caused by exercise, thus supporting the neuroanatomical plasticity of the brain [67,68] (Table 4).

### 4.2. Functional Network Reorganization

**Figure 2:**
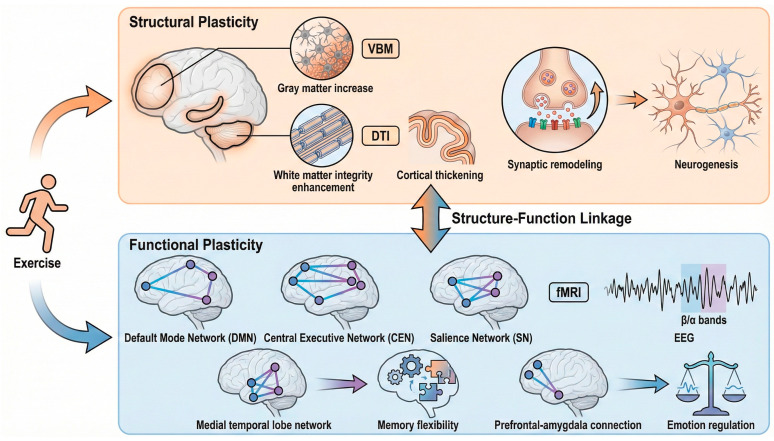
Schematic of exercise-induced brain structural-functional remodeling. The diagram delineates the bidirectional relationship between structural changes (orange panel; e.g., neurogenesis, white matter integrity) and functional network reconfiguration (blue panel; e.g., DMN/CEN dynamics). It highlights how exercise-induced synaptic remodeling underpins the macroscopic shift in brain network efficiency and emotion regulation capacity.

In addition to structural adaptation, exercise triggers dynamic rearrangement of functional brain networks, which streamline cognitive processing and distribution of resources. Neuroimaging research has demonstrated that physical exercise alters the functional connectivity of major large-scale networks, including the default mode network (DMN), central executive network (CEN), and salience network (SN). Exercise enhances the coordination and integration of these networks, which facilitates information processing and attentional control [64]. Mechanistically, we hypothesize that these macro-scale reorganizations are underpinned by a multiscale causal chain starting from molecular triggers. Exercise-induced hippocampal BDNF upregulation likely stimulates the PGC-1α regulatory pathway, facilitating mitochondrial biogenesis and the expression of key synaptic proteins like synaptophysin (SYP) and PSD-95. These cellular adaptations provide the necessary metabolic and structural substrate for the observed changes in network dynamics and connectivity [71,72]. As an illustration, in patients with chronic pain, motor control exercise enhances fractional amplitude of low-frequency fluctuations (fALFF) in the precuneus, one of the central DMN nodes, but decreases the effective connectivity of the dorsolateral prefrontal cortex (DLPFC), one of the main CEN nodes, which is correlated with symptom reduction [73]. On the same note, interventions of aerobic exercise have been observed to enhance functional connectivity of the network of the medial temporal lobe that mediates the enhancement of the mnemonic flexibility and the generalization of learning [74]. Neurotransmitter systems and neuroinflammatory pathways also can be regulated by exercise and could be at the basis of the changes in network dynamics and flexibility [75]. EEG studies have shown electrophysiological evidence that indicates that exercise improves cortical activation and cortico-cortical coupling, which is seen as an increase in phase-locking values and spectral power of the beta and alpha bands, which are signs of increased brain network integration during and after exercise [76,77]. These functional restructurings lead to better executive functions, attention cycle and memory, showing that exercise facilitates the plasticity of a brain network by dynamically reconstituting connectivity patterns to optimize the allocation of cognitive resources and adaptation capacity of behavior (Figure 2).

### 4.3. Differences Between Acute and Chronic Exercise Effects

**Figure 3:**
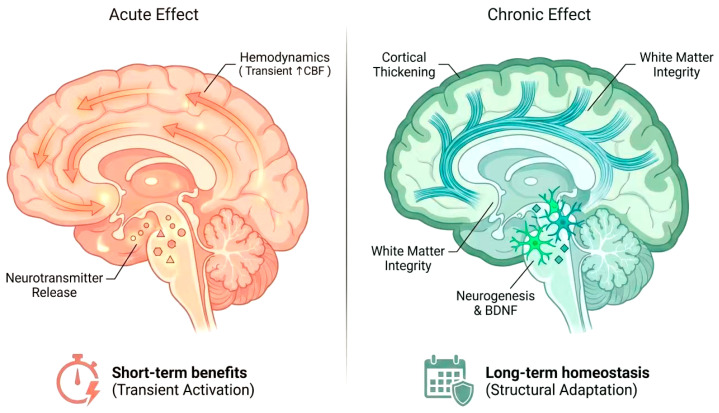
Spatiotemporal dissociation of neural mechanisms between acute and chronic exercise. **Acute Exercise** (**Left Panel**): Characterized by transient functional modulation. The schematic illustrates the immediate hemodynamic response (increased cerebral blood flow, CBF) and the release of neurochemicals (e.g., endocannabinoids) in the hippocampus, leading to short-term cognitive benefits and cortical excitability. The stopwatch icon represents the transient, time-limited nature of these effects. **Chronic Exercise** (**Right Panel**): Characterized by long-term structural adaptation. The illustration depicts morphological changes including cortical thickening, enhanced white matter integrity, and hippocampal neurogenesis (mediated by BDNF upregulation), contributing to long-term brain homeostasis and resilience. The calendar icon signifies the cumulative effect over weeks to years.

The neural consequences of exercise have dual effects on the brain based on their acute and chronic nature. Since the brain is sensitive to changes with time, exercise has two unique time perspectives to its effects [78,79]. The immediate enhancement of neural activity and cognitive performance are found to be temporary and dependent on acute exercise that causes temporary changes with the increase or decrease in cerebral blood flow, metabolism, and release of certain neurotransmitters. As an example, one aerobic exercise action increases the activity of the hippocampus and raises the levels of endocannabinoids, including anandamide, that are associated with better motor sequence memory and hippocampal-associated functions [55,80,81]. Acute exercise also temporarily disinhibits the cortex and increases corticospinal excitement which is further increased during the combination with modalities such as blood flow restriction [82]. These quick adaptations can provide quick cognitive and performance activation but need repetitive stimulation to harden long time adaptations. Conversely, chronic training causes long-term structural and functional changes, such as cortical thickening, white matter integrity and rearrangement of functional networks which uphold enduring cognitive and mood enhancements [70,83]. Neurotrophic factors like BDNF are also upregulated by chronic physical activity, neurogenesis in adult hippocampus is promoted, and neuroplastic changes are normalized in mitochondrial functioning [84]. Notably, changes in brain functions that result in better disease outcomes in neurodegenerative and psychiatric disorders are attributed to the adaptations that occur with chronic exercise stabilizing neural circuits and increasing the resilience of the brain [85]. Mechanistic differences between acute and chronic actions emphasize that exercise prescriptions have to be tailored with regard to intensity, duration, and frequency to maximize short-term and long-term neural outcomes [85,86] (Figure 3, Table 5).

### 4.4. Practical Perspectives: Dose–Response and Precision Prescription

**Figure 4:**
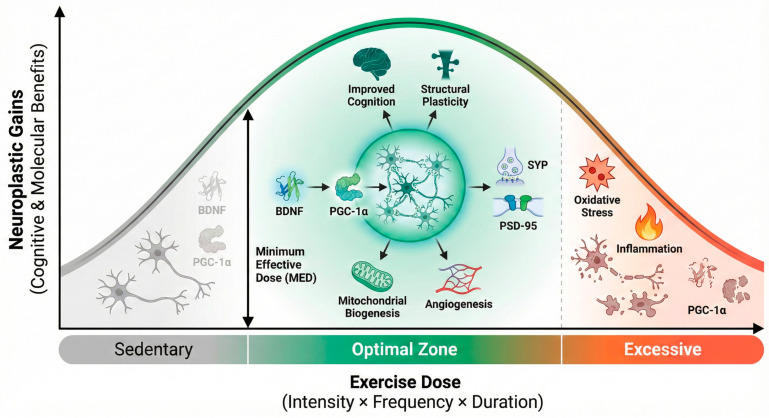
Dose–response relationship between exercise parameters and neuroplasticity gains. The curve illustrates the hypothesized optimal zone for cognitive and molecular benefits, highlighting the minimum effective dose (MED) across intensity, frequency, and duration.

To bridge the gap between multiscale neuroplastic mechanisms and clinical application, understanding the dose–response relationship is essential [91,92]. Current evidence suggests that exercise-induced benefits on cognitive and neural health follow a non-linear pattern, where a “minimum effective dose” (MED) is required to trigger significant molecular and functional adaptations [93,94,95]. As illustrated in Figure 4, moderate-intensity aerobic exercise (e.g., 40–60% VO_2_max) performed for at least 150 min per week across 3 to 5 sessions appears to be the optimal zone for most neuroplastic markers. However, precision intervention must account for individual baseline fitness and “ceiling effects” in high-performing populations to avoid the diminishing returns associated with overtraining. This hierarchical understanding facilitates the transition from generic physical activity recommendations to targeted, multi-target behavioral interventions [91,96,97].

## 5. Exercise-Mediated Enhancement of Cognition, Emotion, and Behavior

### 5.1. Executive Function and Attention Enhancement

Exercising has always been shown to have a great impact on executive functions—including working memory, inhibition, and cognitive flexibility—which are essential for efficient information processing and task switching. Even light intensive acute periods of aerobic exercises have been shown to change cortical excitability and enhance multitasking performance in healthy adults, which is indicated by decreases in response times and increased intracortical facilitation [87,98]. Such advantages of the mind are time-limited; the gains related to acute exercise training are short-term, but the gains associated with long-term exercise training can be longer-lasting [99,100]. Indicatively, moderate-intensity aerobic exercise was reported to enhance Stroop task performance and working memory in adolescents and adults to which neural correlates and localized prefrontal cortex regions engaged in the executive control [100,101]. Resistance exercise also has similar effects on executive functions and attention, occasionally with even stronger effects on attention than aerobic exercise [102]. The integration additive or synergistic effects of combined exercise modalities, combining aerobic with resistance exercise, also yield impressive changes in executive function [62,103,104].

Research on neuroimaging shows that exercise improves the use of the dorsolateral prefrontal cortex (DLPFC) and additional foci of the motor system in executing executive functions, which reflect a better neural efficiency and coordination [99,105]. Moreover, cognitive benefits of exercise depend on individual differences in the cardiorespiratory fitness level and sex hormone differences but the latter seems not to have a great influence on the extent of executive advantages [101,106]. It is also important to note that there are also contexts where exercise would be identified to selectively improve executive functions such as those related to inhibitory control; for instance, exercise conducted in underwater conditions, which highlights the strength of exercise effects in contexts [107,108,109]. Taken together, these results provide support to the idea that physical exercise, specifically at moderate intensity and lasting in the long term, strongly improves the executive functions and attention mediated by the improved performance of the prefrontal cortex and the better organization of neural network functions [110,111].

### 5.2. Emotion Regulation and Mental Health Promotion

Exercise has far-reaching regulatory clinical consequences on emotional procedures and mental wellbeing, mainly by achieving control of neuroendocrine systems and emotion-regulating brain circuits [59,112]. Among them attenuation of the hypothalamic–pituitary–adrenal (HPA) axis activity due to exercise leads to a decrease in circulating stress hormones, including cortisol, which in turn relieves anxiety and depressive symptoms [113]. Exercise-based behavior change models have been shown to produce substantial changes in psychological outcome measures such as depression, anxiety, and hostility, and increased wwellbeingand emotional regulation self efficacy in diverse groups of people, such as college students and people with mental health conditions [114,115,116]. Neuroimaging and electrophysiological examinations demonstrate that exercise boosts prefrontal cortex activity and also functional association with limbic areas including the amygdala which enables them to better perceive, value and regulate emotional reactions [89,113]. Another neural correlate of emotion regulation, which is also affected by exercise, is frontal alpha asymmetry, which is associated with high anxiety or with test anxiety, and is something that indicates a stronger top-down regulation of negative affect [88,117]. Tai Chi and mindfulness-based interventions are some of the mind–body exercise modalities which enhance working memory and ability to manage emotions, which further sustain the benefits of exercise in psychological resiliency [118,119]. Furthermore, evidence suggests that exercise interventions can decrease implicit negative affect and concurrently promote adaptive emotion regulation strategies in clinical populations, such as patients with personality disorders [90]. The fact that self-efficacy and mindfulness mediate the association between physical exercise and subjective wellbeing highlights the role of psychological processes in the functionality of exercise in mental wellbeing [120,121]. Notably, the way mental health professionals promote exercise is suboptimal, which indicates that psychiatrists require more training and exercise integration into psychotherapy should be emphasized [25,122]. Altogether, exercise enhances emotion management and mental health through neuroendocrine-mediation, prefrontal-limbic-circuitry-enrichment, and psychological-empowerment and can be used as a strong adjunctive approach in promoting mental health [123].

### 5.3. Behavioral Adaptation and Potential for Addiction Intervention

**Figure 5:**
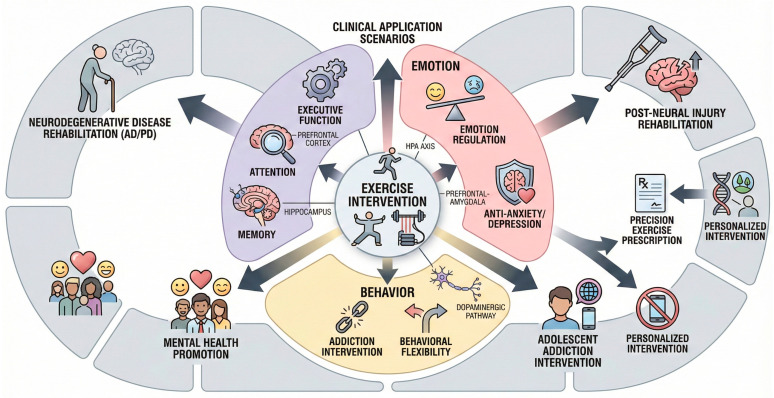
Panorama of cognitive-behavioral-clinical translational applications of exercise. Figure 5 centered on “exercise intervention” with three core effect modules (light purple: cognition; light pink: emotion; light yellow: behavior) radiating outward. Outer ring (light gray) for clinical scenarios; dark gray arrows indicate translation.

Exercise interventions have demonstrated indicative effectiveness in factoring maladaptive behaviors and alleviating the aspect of addictiveness, encompassing current behavioral addiction phenotypes like smartphone and internet addiction. Exercise decreases compulsive and maladaptive activities of exercise per se, which suggests that exercise contributes to behavioral flexibility [124,125]. In particular, exercising reduces smartphone addiction and internet addiction symptoms in adolescents and young adults, and diverse ones such as aerobic exercise, mindfulness, badminton, and Tai Chi have been shown to have considerable advantages [54,126]. Meta-analyses validate that exercise interventions can produce remarkable addiction scores and associated psychological symptoms, longer-lasting and moderate-intensity exercise interventions produce better results [127]. Exercise also enhances executive functions and self-control abilities of resisting addictive behaviors, as both are neurobiologically mediated by the reward system and heightened emotion regulation [128,129]. Exercise in substance use disorder reduces drug-seeking behavior and withdrawal symptoms which may occur through neuroplasticity in dopaminergic pathways and expression of neurotrophic factors [130,131]. Moreover, exercise enhances the mental health comorbidities in the recovery process which can regulate the mental disorders that go hand in hand with addiction, like depression and anxiety [132,133]. There are psychological factors such as obsessive-compulsive personality and self-efficacy which interplay with the risk of the addiction to exercise, which implies that interventions aimed at mitigating based on these aspects can maximize gains [134]. All in all, exercise will be considered a multifaceted intervention capable of behavioral adaptation as well as reduce addictive behaviors through increasing cognitive control, regulating the reward circuit, and improving emotional control (Figure 5, Table 6).

## 6. Conclusions and Perspectives

In summary, we have further elucidated the multifacetedness of the action effects on neuroplasticity and mental and psychological health of physical exercise in the context of a multiscale including the molecular, cellular, brain network and behavioral pathways. Proficiently, this review highlights the manner in which neurotrophic factor alterations, metabolic alterations, and neurotransmitter processes, which are the product of exercise, form the molecular framework that establishes the foundation of remodeling of the brain structure and functioning. The neural changes are characterized at the networks level which, at the ultimate outcome, are underscored in the rearrangement of the brain circuits and, therefore, enhanced intellectual performance and behavioral adaptability. Such a framework presents the richness of the consequences of exercise and demonstrates that an integrative approach is needed to be able to observe the full potential of exercise as a method of therapy [36].

Coming to a consensus between various visions of the study, it must be said that the individual differences play a significant part in the mediation of the exercise outcomes. The importance of physical activity responsiveness is due in large part to genetic predispositions, developmental stages, and socio-environmental backgrounds, providing a noteworthy incentive to individualize the prescription of exercise. This critical understanding is a problem of the one-size-fits-all-paradigm and it requires special interventions that understand the unique situations of the biology of a person and his or her situations to achieve the optimal neurocognitive outcomes [136,137].

Integration of novel approaches like multimodal neuroimaging and computational modeling along with the discovery of biomarkers have propelled the field of precision exercise medicine. They enable the breaking down of complex brain-behavior relationships and they enable the development of evidence-based interventions. Moreover, the translational potential of such integrative solutions is not restricted to the limits of the research only and extends to the scope of the practice of clinical and educational implementation, where customized exercise programs may be used to induce neurodevelopment, cognitive rehabilitation and mental health maintenance.

The further research must involve a more rigid account of the cause–effect process between some exercise modalities and doses with specific neurobiological and functional outcomes [138]. The emergence of interdisciplinary collaborative platforms will also be required to value the knowledge in neuroscience, physiology, psychology, and data science and advance the innovative research paradigms [139]. Also, considering that exercise is a pharmacological-like behavioral intervention approach [140], it is notable that this knowledge should be translated into policy frameworks which should aim to encourage its use and accessibility to all [141]. Such policies are expected to advance exercise as a form of frontline intervention either by prevention or treatment doctrines because it is cheap and also has minimal side effects [142].

**Figure 6:**
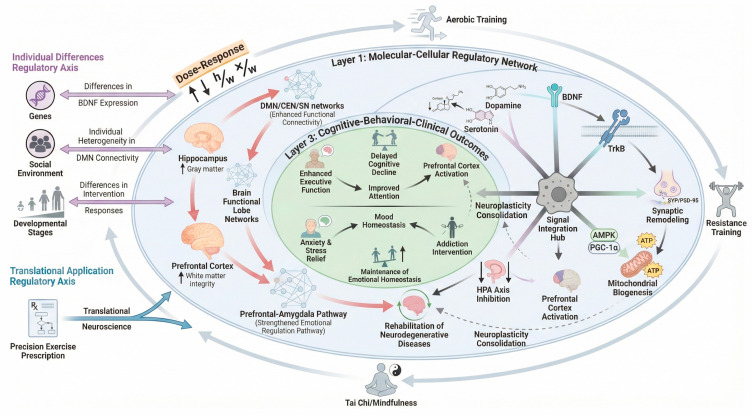
The multiscale integrative regulatory network of exercise-induced neuroplasticity. This schematic illustrates the hierarchical integration of exercise effects across three biological scales: (Layer 1) Molecular-Cellular Regulatory Network: exercise triggers the release of neurotrophic factors (e.g., BDNF, IGF-1), enhances mitochondrial biogenesis (PGC-1α pathway), and modulates synaptic plasticity. (Layer 2) Brain Structure and Functional Networks: these molecular signals drive macroscopic adaptations, including white matter integrity, cortical thickening, and the reorganization of large-scale networks (DMN, CEN, SN). (Layer 3) Cognitive-Behavioral-Clinical Outcomes: the cumulative effects lead to observable benefits such as enhanced executive function, emotional homeostasis, and neuroprotection against degeneration. Regulatory Axes: the model is modulated by individual differences (left axis; e.g., genetics, environment) and informs precision exercise prescription (right axis; e.g., dose–response, translational application), highlighting the closed-loop nature of neuroplasticity consolidation.

Comprehensively, this review confirms that the power of physical exercise as a modulator of brain plasticity and cognitive wellbeing through multifaceted and compartmentalized processes [143]. However, the field of exercise-induced neuroplasticity must now address three grand challenges to move toward transformative scientific synthesis. First, a significant translational gap remains in verifying whether molecular signaling pathways discovered in rodent models—such as specific myokine–brain crosstalk—fully account for clinical neuroplasticity in humans. Second, there is a critical need for personalized exercise prescriptions that account for individual variability in genetics and environmental stressors. Finally, the scalability of these interventions into real-world public health frameworks remains a prerequisite for maximizing the therapeutic potential of physical exercise as a ‘multi-target’ intervention. When the role of molecular understanding is combined with the information at the network level and behavioral domain, and even the variation in the individual is taken into account, the field will enter the next stage and ** move toward the goal of ** precision exercise intervention [144,145] (Figure 6). Additional research on non-specific areas, such as the works of science and supporting policies will take a pivotal role in adapting these scientific findings into the actual use of real life experiences [146], which in the long-term will enhance human brain capabilities and mental security [147].

## Figures and Tables

**Table 1:** Key neurotrophic factors in exercise-induced synaptic plasticity.

Neurotrophic Factors	Core Signaling Pathways	Major Biological Functions	Related Diseases/Model Applications	References
Brain-Derived Neurotrophic Factor (BDNF)	BDNF–TrkB-MAPK/PI3K/Akt	Activates long-term potentiation (LTP) and regulates synaptic strength; promotes the expression of synaptic proteins (SYP, PSD-95); supports neurogenesis and neuronal survival	Ischemic stroke, Mild Cognitive Impairment (MCI), Alzheimer’s Disease (AD), Glaucoma, Depression	[22,23,27,37]
Insulin-Like Growth Factor-1 (IGF-1)	Metabolic Regulation Pathways	Synergizes angiogenesis and metabolic support; improves age-related and disease-related cognitive functions; maintains synaptic stability	Parkinson’s Disease (PD), Stroke, Normal Aging	[24,25,29]
Vascular Endothelial Growth Factor (VEGF)	Angiogenic Pathways	Enhances cerebral blood flow supply; improves brain metabolic adaptability; synergizes with BDNF/IGF-1 to promote neuroplasticity	MCI, stroke, neurodegenerative diseases	[25,26]
Glial Cell Line-Derived Neurotrophic Factor (GDNF)	Neurotrophic Signaling Pathways	Participates in neuroprotection in PD patients; regulates synaptic plasticity; alleviates functional disorders after nerve injury	PD, traumatic brain injury (TBI)	[29]

Note: “neurotrophic factors and synaptic plasticity” corresponds to the third paragraph elaboration on BDNF–TrkB pathway activation of MAPK and PI3K/Akt pathways, the third paragraph regulation of synaptic plasticity markers by BDNF in ischemic stroke models, and the sixth paragraph research on exercise-induced upregulation of synaptic proteins.

**Table 2:** Exercise-induced mitochondrial and metabolic adaptations in the brain.

Adaptation Types	Key Regulatory Factors/Pathways	Core Metabolic Changes	Physiological Significance and Pathological Associations	References
Mitochondrial Biogenesis	AMPK/PGC-1α Pathway	Promotes mitochondrial proliferation; maintains membrane potential and integrity; inhibits swelling and apoptosis	Resists stress such as chemotherapy and ischemia; improves mitochondrial dysfunction in diabetes and neurodegenerative diseases	[37,40,45]
Enhancement of Energy Metabolism Efficiency	Glycolysis/Oxidative Phosphorylation Pathways	Enhances TCA intermediates; optimizes amino/fatty acid metabolism	Meets the energy demand of neurons during exercise; improves fatigue resistance	[41,43]
Oxidative Stress Resistance	Antioxidant Signaling Pathways	Enhances brain antioxidant capacity; reduces ROS oxidative damage	Delays neurodegenerative processes; improves oxidative stress imbalance in mood disorders	[45,46]
Mitochondrial Membrane Function Regulation	Mitochondrial Connexin 43	Mediates mitochondrial stress adaptation; enhances stability; regulates mito-cytoplasm signaling	Provides new molecular targets for exercise-induced mitochondrial plasticity; associated with neural cell survival	[42]

Note: “Metabolic and Mitochondrial Adaptations” corresponds to the second paragraph regulation of mitochondrial biogenesis by AMPK and PGC-1α, the third paragraph (protection of mitochondrial integrity by exercise in chemotherapy models, and the third paragraph mediating role of mitochondrial connexin 43.

**Table 3:** Exercise-induced changes in neurotransmitter systems and stratified behavioral evidence.

Neurotransmitter Systems	Core Exercise-Induced Changes	Key Brain Regions Involved	Preclinical Evidence (Animal Models)	Clinical Evidence (Human Studies)	References
Dopaminergic System	Increases dopamine release; upregulates D1/D2 receptors; modulates reward circuitry.	Nucleus accumbens, striatum, hippocampus	Reduces drug-seeking behaviors and cravings in addiction models; enhances spatial memory.	Improves executive function and motivation; enhances motor learning consolidation.	[25,47,48]
Serotonergic System	Increases serotonin synthesis; normalizes SERT expression; activates 5-HT1A/2A receptors.	Hippocampus, dorsal raphe nucleus	Promotes hippocampal neurogenesis; reduces anxiety-like behaviors in stress models.	Self-reported reduction in anxiety symptoms; improved mood regulation scores.	[30,50]
Glutamatergic System	Balances excitatory-inhibitory signaling; regulates NMDA/AMPA receptor subunits.	Cerebral cortex, hippocampus	Enhances long-term potentiation (LTP); improves synaptic plasticity markers.	Improves cognitive performance in complex tasks; supports learning and memory retention.	[41]
Endocannabinoid System	Increases anandamide (AEA) levels; modulates synaptic transmission efficiency.	Hippocampus, cerebral cortex	Mediates anxiolytic effects via CB1 receptors; facilitates extinction of fear memories.	Acute increases in serum AEA correlate with improved motor sequence memory and mood.	[55]

Note: Data are stratified into preclinical evidence (derived primarily from rodent models) and clinical evidence (derived from human trials) to explicitly distinguish mechanistic insights from translational outcomes. Abbreviations: 5-HT: 5-hydroxytryptamine; SERT: serotonin transporter; AEA: anandamide; CB1: cannabinoid receptor type 1.

**Table 4:** Exercise-induced structural plasticity of the brain.

Types of Structural Plasticity	Detection Techniques	Major Affected Brain Regions	Physiological/Pathological Effects	References
Increase in Gray Matter Volume/Density	Voxel-Based Morphometry (VBM)	Hippocampus, temporal lobe, occipitotemporal cortex (fusiform gyrus, lateral occipital cortex)	Enhances memory function; improves visuomotor integration; prevents neurodegenerative atrophy	[62,69]
Improvement of White Matter Integrity	Diffusion Tensor Imaging (DTI)	Prefrontal cortex, cerebellar nerve fiber tracts	Enhances neural fiber connectivity; stabilizes neural networks; improves executive control and memory consolidation	[64]
Cortical Thickening	Structural Magnetic Resonance Imaging (sMRI)	Motor cortex, temporal cortex	Promotes motor skill learning; improves functional recovery after peripheral nerve injury	[65,70]
Synaptic Structural Remodeling	Transcranial Magnetic Stimulation (TMS), histological analysis	Hippocampus, cerebral cortex synapses	Increases synaptic number and strength; improves synaptic plasticity in MCI patients; supports neural repair	[26,66]
Resistance to Gray Matter Atrophy	Structural Magnetic Resonance Imaging (sMRI)	Global gray matter regions	Prevents temporary gray matter loss under stress (e.g., Antarctic isolation)	[9]

Note: “Structural Brain Changes” corresponds to the first paragraph’s classic study on exercise-induced hippocampal volume increase, the fourth paragraph modulation of brain structure by treadmill exercise after peripheral nerve injury, and the first paragraph protection of gray matter by exercise in Antarctic isolation environments.

**Table 5:** Acute and chronic exercise effects on brain function and behavioral outcomes.

Exercise Types	Core Changes in Brain Function	Behavioral Outcomes	Duration of Effects	References
Acute Exercise	Enhanced hippocampal activity; cortical disinhibition; increased beta/alpha band spectral power; elevated endocannabinoid levels	Short-term improvement in motor sequence memory and multitasking ability; relief of acute anxiety; enhanced attention	Hours to days	[55,82,87]
Acute Exercise	Modulation of prefrontal cortex excitability; increased corticospinal excitability	Improved emotion regulation in test-anxious individuals; enhanced Stroop task performance	Hours	[76,88]
Chronic Exercise	Optimized functional connectivity of Default mode network (DMN)/central executive network (CEN); sustained upregulation of BDNF	Long-term improvement in memory flexibility and learning generalization; delayed cognitive decline; stable emotion regulation	Weeks to months	[64,74,84]
Chronic Exercise	Increased dynamic flexibility of medial temporal lobe network; normalized mitochondrial function; enhanced neurogenesis	Improved symptoms of PD/AD; reduced risk of addiction relapse; enhanced psychological resilience	Months to years	[83,85]
Chronic Exercise	Enhanced prefrontal-amygdala functional connectivity; regulation of frontal alpha asymmetry	Relief of depression/anxiety symptoms; improved emotion regulation strategies in patients with personality disorders	Weeks to months	[89,90]

Note: “Differences Between Acute and Chronic Exercise Effects” and “Emotion Regulation and Mental Health Promotion”correspond to second paragraph on acute exercise and motor sequence memory; the third paragraph on the improvement of neurodegenerative diseases by chronic exercise, and the fifth paragraph on the effects of exercise on emotion regulation in patients with personality disorders.

**Table 6:** Recommended Exercise Regimens for Diverse Intervention Areas.

Intervention Areas	Recommended Exercise Regimens	Core Mechanisms of Action	Target Populations/Applicable Scenarios	References
Cognitive Enhancement (Executive Function/Attention)	Moderate-Intensity Aerobic Exercise Resistance Exercise (Combined Mode)	Activates prefrontal cortex and motor system; enhances neural efficiency; improves cortical excitability and synaptic plasticity	Healthy adults, adolescents, mci patients, middle-aged women	[24,62,102]
Emotion Regulation and Mental Health Promotion	Aerobic Exercise Tai Chi Mindfulness Exercise	Inhibits hypothalamic–pituitary–adrenal (HPA) axis; reduces cortisol levels; enhances prefrontal-limbic circuitry connectivity	College students, patients with depression/anxiety, test-anxious individuals, Patients with Personality Disorders	[88,113,118]
Addiction Intervention	Aerobic Exercise (Jogging/Swimming) Badminton Tai Chi	Regulates dopaminergic pathway neuroplasticity; enhances executive control and self-control; improves comorbid mood disorders	Adolescents/young adults (smartphone/internet addiction), patients with substance use disorders	[24,126,135]
Neurodegenerative Disease Rehabilitation	Task-Oriented Training Dance Exercise Aerobic Exercise	Increases BDNF/IGF-1 levels; maintains brain structural integrity; improves mitochondrial function; enhances motor function	AD patients, PD patients, MCI patients, elderly population	[18,70,83]
Cognitive Rehabilitation After Neural Injury	Moderate-Intensity Aerobic Exercise Interval Training	Enhances brain network functional connectivity; promotes synaptic remodeling; improves brain metabolic homeostasis	Post-stroke patients, patients with peripheral nerve injury, individuals with subjective cognitive decline	[13,26,65]

Note: This table’s references are distributed across exercise intervention-related studies and refer to the acute effects of combined exercise on executive function, exercise intervention for adolescent internet addiction, and the influence of individual factors on exercise intervention effectiveness.

## Data Availability

No new data were created or analyzed in this study. Data sharing is not applicable to this article.

## Abbreviations

The following abbreviations are used in this manuscript:

BDNF	Multidisciplinary Digital Publishing Institute
IGF-1J	Directory of open access journals
VEGF	Vascular Endothelial Growth Factor
GDNF	Linear dichroism
LTP	Long-Term Potentiation
MCI	Mild Cognitive Impairment
AD	Alzheimer’s Disease
PD	Parkinson’s Disease
TBI	Traumatic Brain Injury
AMPK	AMP-activated Protein Kinase
PGC-1α	Peroxisome Proliferator-Activated Receptor Gamma Coactivator 1-alpha
ROS	Reactive Oxygen Species
SERT	Serotonin Transporter
5-HT	5-Hydroxytryptamine (Serotonin)
DMN	Default Mode Network
CEN	Central Executive Network
SN	Salience Network
fALFF	Fractional Amplitude of Low-Frequency Fluctuations
DLPFC	Dorsolateral Prefrontal Cortex
EEG	Electroencephalography
VBM	Voxel-Based Morphometry
DTI	Diffusion Tensor Imaging
sMRI	Structural Magnetic Resonance Imaging
TMS	Transcranial Magnetic Stimulation
HPA	Hypothalamic–Pituitary–Adrenal Axis
AEA	Anandamide
